# Wearable **Augmented Reality** Platform for Aiding Complex 3D Trajectory Tracing

**DOI:** 10.3390/s20061612

**Published:** 2020-03-13

**Authors:** Sara Condino, Benish Fida, Marina Carbone, Laura Cercenelli, Giovanni Badiali, Vincenzo Ferrari, Fabrizio Cutolo

**Affiliations:** 1 Information Engineering Department, University of Pisa, 56126 Pisa, Italy; fida.benish@dii.unipi.it (B.F.); marina.carbone@endocas.unipi.it (M.C.); vincenzo.ferrari@unipi.it (V.F.); 2 Maxillofacial Surgery Unit, Department of Biomedical and Neuromotor Sciences and S. Orsola-Malpighi Hospital, Alma Mater Studiorum University of Bologna, 40138 Bologna, Italy; laura.cercenelli@unibo.it (L.C.); giovanni.badiali@unibo.it (G.B.)

**Keywords:** visual augmented reality, optical tracking, head-mounted display, video see-through, 3D trajectory tracing

## Abstract

Augmented reality (AR) Head-Mounted Displays (HMDs) are emerging as the most efficient output medium to support manual tasks performed under direct vision. Despite that, technological and human-factor limitations still hinder their routine use for aiding high-precision manual tasks in the peripersonal space. To overcome such limitations, in this work, we show the results of a user study aimed to validate qualitatively and quantitatively a recently developed AR platform specifically conceived for guiding complex 3D trajectory tracing tasks. The AR platform comprises a new-concept AR video see-through (VST) HMD and a dedicated software framework for the effective deployment of the AR application. In the experiments, the subjects were asked to perform 3D trajectory tracing tasks on 3D-printed replica of planar structures or more elaborated bony anatomies. The accuracy of the trajectories traced by the subjects was evaluated by using templates designed ad hoc to match the surface of the phantoms. The quantitative results suggest that the AR platform could be used to guide high-precision tasks: on average more than 94% of the traced trajectories stayed within an error margin lower than 1 mm. The results confirm that the proposed AR platform will boost the profitable adoption of AR HMDs to guide high precision manual tasks in the peripersonal space.

## 1. Introduction

Visual Augmented Reality (AR) technology supplements the user’s perception of the surrounding environment by overlaying contextually relevant computer-generated elements on it so that the real world and the digital elements appear to coexist [1,2]. Particularly in visual AR, the locational coherence between the real and the virtual elements is paramount to supplementing the user’s perception of and interaction with the surrounding space [3]. Published research provides glimpses of how AR could dramatically change the way we learn and work, allowing the development of new training paradigms and efficient means to assist/guide manual tasks.

AR has proven to be a key asset and an enabling technology within the fourth industrial revolution (i.e., Industry 4.0) [4]. A large number of successful demonstrations have been reported in maintenance and repair tasks through instructions with textual, visual, or auditory information [5,6,7]. AR is capable to dramatically reduce the operators learning curve in performing complex assembly sequences [8,9,10,11] and in improving the overall process task [12].

Similarly, one of AR’s most common applications is during stages related to product manufacturing [13,14,15,16], aimed to increase productivity and time-efficiency compared to standard instruction media such as paper manuals and computer terminals.

AR technology allows the user to move and interact with the augmented scene removing the need to shift attention between the digital instructions and the actual environment [9]. Published works show evidence of improved performance efficiency, time to task completion, and mental workload [17].

Head-Mounted Displays (HMDs) are emerging as the most efficient output medium to support complex manual tasks performed under direct vision (e.g., in surgery). This is owing to their ability to preserve the user’s egocentric perception of the augmented workspace and so allow the hands-free interaction with it [18,19]. The growing availability of consumer level Optical See-Through (OST) HMDs has stimulated a burgeoning market for a broad range of potential AR applications in education and training, healthcare, industrial maintenance, and manufacturing. However, extensive research is still needed to develop a robust, untethered, power-efficient, and comfortable headset, acting as a “*transparent interface between the user and the environment—a personal and mobile window that fully integrates real and virtual information*” [20].

One of the largest obstacles to the successful adoption of the existing technology for guiding high precision manual tasks is the inability to render proper focus cues: indeed, the majority of commercial HMD systems offer the AR content at a fixed focal distance outside the peripersonal space (>1 m), thus failing to stimulate natural eye accommodation and retinal blur effects. Recent research studies show that this not only leads to visual fatigue, but also to a proven reduction in user performance in completing a task, which requires keeping both the real and the virtual information in focus simultaneously, for example, to integrate virtual and real information for a reading task [21], or to connect points with a line [22].

The lack of perceptual conflicts, the accurate calibration mechanisms, system ergonomics, and low latency are the basic requirements that an AR headset should comply in order to be used as a reliable aid to high-precision manual tasks such as in surgical or industrial applications [23].

These requirements have been recently translated into a working prototype of a new-concept AR headset developed within the European project VOSTARS (Video and Optical See-Through Augmented Reality Surgical Systems, Project ID: 731974 [24]). The overarching goal of the project is to design and develop a new-concept wearable AR platform capable of deploying both video and optical see-through-based augmentations for the peripersonal space and to validate it as tool for surgical guidance.

The AR platform comprising the software framework and an early version of the custom-made HMD were thoroughly described in a recently published paper [23]. In the work, the results of an experimental study aimed at assessing the efficacy of the AR platform in guiding a simulated task of tissue incision were also presented.

These results were very encouraging and they supported the claim that the wearable AR framework could represent an effective tool in guiding high-precision manual tasks.

Along the same line of reasoning, with this work, we show the results of a user study whose goal is to provide a conclusive answer as to whether the AR platform under video see-through (VST) modality can be an effective tool in guiding complex 3D trajectory tracing tasks on 3D-printed replica of planar structures or more elaborated bony anatomies.

## 2. Materials and Methods

**Figure 1:**
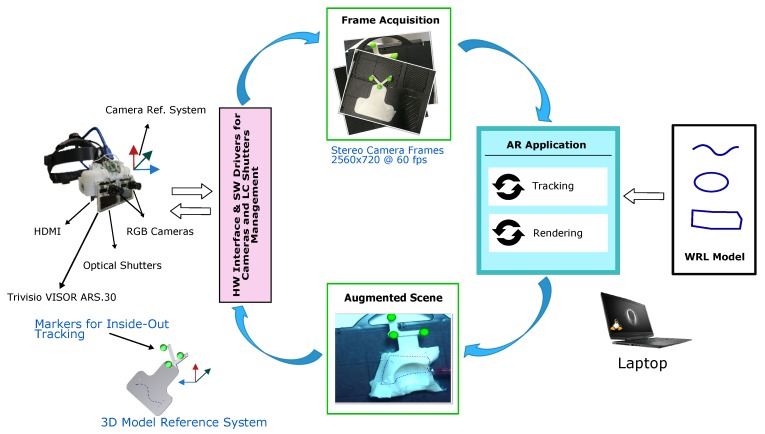
Overview of the hardware and software components of the wearable Augmented Reality (AR) platform for aiding high-precision manual tasks in the peripersonal space. The AR framework runs on a single workstation (i.e., a laptop) and can implement both the optical see-through (OST) and the video see-through (VST) mechanisms.

This section provides a detailed description of the hardware and software components. All components are depicted in Figure 1.

### 2.1. Custom-Made Head-Mounted Display

The custom-made hybrid video-optical see-through HMD fulfills strict technological and human-factor requirements towards the realization of a functional and reliable aiding tool for high-precision manual tasks. The HMD was assembled by re-working and re-engineering a commercial OST visor (ARS.30 by Trivisio [25]).

As described in [23], the key features of the HMD were established with the aim of mitigating relevant perceptual conflicts typical of commercial AR headsets for close-up activities.

Notably, the collimation optics of the display were re-engineered to offer a focal length of about 45 cm, which constitutes, when used for close-up works, a defining and original feature to mitigate the vergence-accommodation conflict and the focus rivalry. The HMD was incorporated in a 3D printed plastic frame together with a pair of Liquid Crystal shutters and a pair of front-facing USB 3.0 RGB cameras [26]. The stereo camera pair is composed by two LI-OV4689 cameras by Leopard Imaging, both equipped with 1/3″ OmniVision CMOS 4M pixels sensor (pixel size of 2 μm). The cameras were mounted with an anthropometric interaxial distance (∼6.3 cm) and with a fixed convergence angle. In this way, we could ensure sufficient stereo overlap at about 40 cm (i.e., an average working distance for manual tasks). Both the cameras are equipped with an M12 lens support whose focal length (f = 6 mm) was chosen to compensate for the zoom factor due to the eye-to-camera parallax along the display optical axis (at ≈40 cm).

The computing unit is a Laptop PC with the following specifications: Intel Core i7-8750H CPU @ 2.20 GHz with 12 cores and 16 GB RAM (Intel Corp., Santa Clara, CA, USA). Graphic card processing unit (GPU) is a Nvidia GeForce RTX 2060 (6GB) with 1920 CUDA Cores (Nvidia Corp., Santa Clara, CA, USA).

### 2.2. AR Software Framework

The software framework is conceived for the deployment of VST and OST AR applications able to support in situ visualization of medical imaging data and specifically suited for stereoscopic AR headsets; the key function of the software, under VST modality, is to process and augment the images grabbed by the stereo pair of RGB cameras before they are rendered to the two microdisplays of the visor. The grabbed frames of the real scene are processed to perform a marker-based optical tracking, which requires the identification of the 3D position of the markers both in the target reference frame and camera reference frame.

The augmented scene is generated by merging the real camera frames with the virtual content (e.g., in our application, the planned trajectories) ensuring the proper locational realism. To accomplish this task, the projection parameters of the virtual viewpoints are set equal to those of the real cameras and the pose of the tracked object defines the pose of the virtual content in the scene [27].

The main features of the software framework can be summarized as follows [23]:

- The software is capable of supporting the deployment of AR applications on different commercial and custom-made headsets.
- The CUDA-based architecture of the software framework makes it computationally efficient.
- The software provides in situ visualization of task-oriented digital content.
- The software framework is highly configurable in terms of rendering and tracking capabilities.
- The software can deliver both optical and video see-through-based augmentations.
- The software features a robust optical self-tracking mechanism (i.e., inside-out tracking) that relies on the stereo localization of a set of spherical markers.
- The AR application achieves an average frame rate of ≈30 fps.

### 2.3. AR Task: Design of Virtual and Real Content

Three trajectories with different degrees of complexity were implemented to test the system accuracy:

1. A 2D curve (79 mm in length) (T1).
2. A 3D curve (130 mm in length) describing a closed trajectory on a convex surface (T2).
3. A 3D curve (223 mm in length) describing a closed trajectory consisting of a series of four curves on concave and convex surfaces (T3).

T1 was designed to test the system on a simple planar phantom simulating, for instance, an industrial manufacturing process that requires cutting flat parts to specific shapes; T2 and T3 were drawn on two anatomical surfaces (i.e., a portion of skull and of acetabulum), and they simulate complex surgical incision tasks.

**Figure 2:**
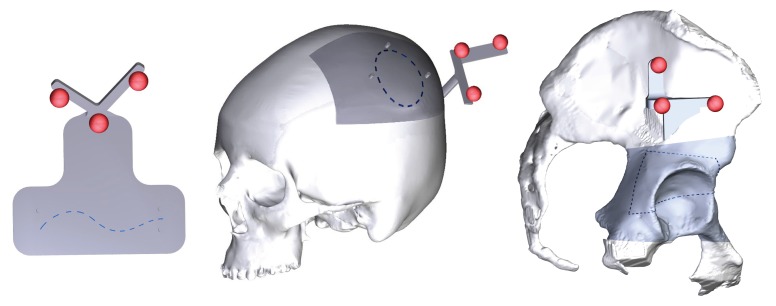
The three trajectories designed for the AR platform evaluation. From the left to the right: T1 over the top side of a rectangular plate, T2 on the surface of a patient-specific skull model, T3 on the surface of a patient-specific acetabular model.

Creo Parametric software was used to design the three trajectories (Figure 2). T1 was drawn on the top side of rectangular plate (size 10 × 5 mm); T2 and T3 were modeled with spline curves by selecting 3D points on a portion of the selected anatomical surface (dark grey portions in Figure 2). The 3D model of the skull and of the acetabulum were generated from real computed tomography datasets, segmented with a semi-automatic segmentation pipeline [28] to extract the cranial and the acetabular bones. A 3D printer (Dimension Elite) was used to turn the phantom virtual models into tangible replicas made of acrylonitrile butadiene styrene (ABS).

The three trajectories were represented with dashed curves (0.5 mm thickness) and saved as .wrl models to be imported by the software framework and displayed as the virtual content of the AR scene.

As previously mentioned, the accurate AR overlay of the virtual trajectory to the physical 3D-printed models is achieved by means of a tracking modality that relies on the real-time localization of three reference markers; for this reason, three spherical markers (11 mm in diameter) were embedded in the CAD model of phantoms as shown in Figure 2. The markers were dyed in fluorescent green, to boost the response of the camera sensor and improve the robustness of the blob detection under uncontrolled lighting conditions [23,29].

### 2.4. Subjects

Ten subjects, 3 males and 7 females, with normal visual acuity or corrected-to-normal visual acuity (with the aid of contact lenses) were recruited from technical employees and University students. Table 1 reports the demographics of the participants included in this study, which were aged between 42 and 25. Participants were asked to rate their experience with AR technologies, HMDs, and VST-HMDs to get the opportunity to correlate these with their performance and subjective evaluation of the AR platform.

### 2.5. Protocol of the Study

**Figure 3:**
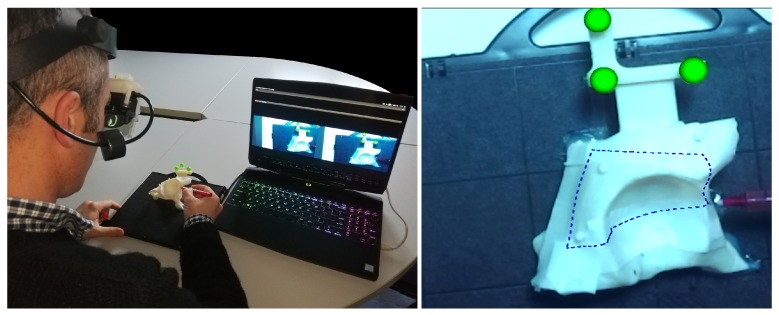
On the left: subject during a T3 task. On the right: AR scene visualized by the subject.

The experimental setting is shown in Figure 3. During the performance of the task, each subject was seated in a chair adjustable in height, at a comfortable distance from the three phantoms and he/she was free to move freely.

The subject was asked to perform the “trajectory tracing” task three times for each trajectory, and to report any perceptible spatial jitter or drift from the AR content. The trajectories were administered in random order.

**Figure 4:**
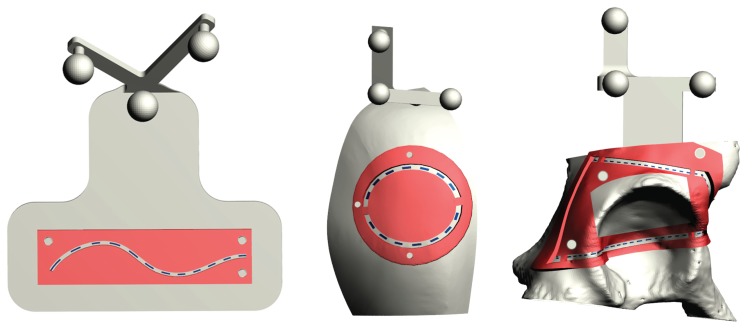
CAD model of the templates designed ad hoc for each phantom to test the accuracy of the trajectories traced by the subjects.

The accuracy of the trajectories traced by the subjects was evaluated by using templates designed ad hoc to match the surface of the phantoms (Figure 4). The templates were provided with inspection windows shaped as the ideal trajectories (dotted blue line in Figure 4), and with engagement holes to ensure a unique and stable placement of the template over the corresponding phantoms.

Three templates were designed for each phantom, with different wide inspection windows, to evaluate three different levels of accuracy: given that the virtual trajectory, as well as the pencil line, have a 0.5 mm thickness, inspection windows measuring 1.5 mm, 2.5 mm, and 4.5 mm in width were designed to test a 0.5 mm, 1 mm, and 2 mm of accuracy level, respectively. We considered as successful only those trials in which the accuracy was ≤2 mm. Indeed, 1–2 mm accuracy is regarded as an acceptable range in many complex manual tasks such as in the context of image-guided surgery [30]. When the traced trajectory was outside the template with thicker inspection window (i.e., the 4.5 mm window), the test was considered as failed.

**Figure 5:**
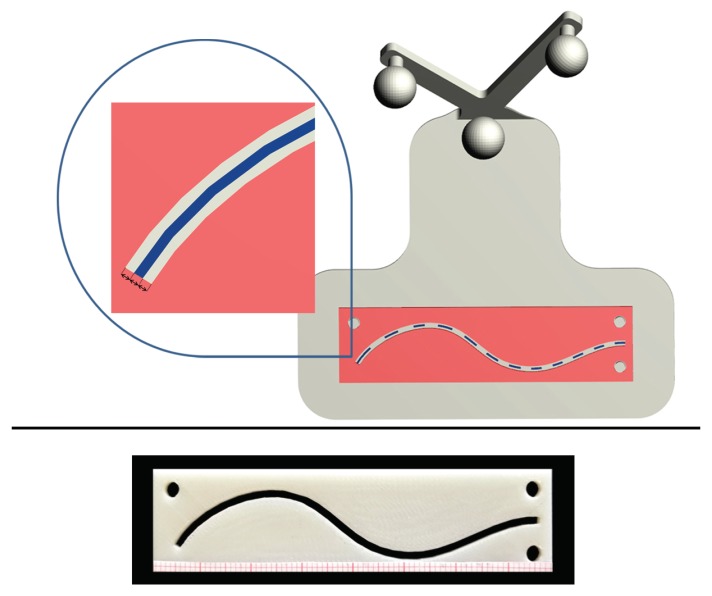
Top: CAD model of the 0.5 mm accuracy level template designed for T1, with a zoomed detail of the inspection window (1.5 mm in width). Bottom: 3D printed template with stripes of graph paper to estimate the cumulative length of the traced trajectory within the inspection window.

In the experiments, stripes of graph paper were used to estimate the cumulative length of the traced trajectory within the inspection windows (Figure 5). In this way, we could estimate the percentage of the traced trajectory staying within the specific accuracy level dictated by the template (Figure 5).

A 0.5 mm pencil was used to draw the perceived trajectory on a masking tape applied over the phantom surface; the tape was removed and replaced at the end of each trial after the evaluation of the user performance.

Subjects were instructed that the primary goal of the test was to accurately trace the trajectories as indicated by the AR guidance; time to completion in tracing the trajectory was recorded using a stopwatch. At the end of the experimental session, subjects were administered a 5-point Likert questionnaire to qualitatively evaluate the AR experience (Table 2).

### 2.6. Statistical Analysis

The SPSS Statistics Base 19 software was used to perform statistical analysis of data. Results of the Likert questionnaire were summarized in terms of median with dispersion measured by interquartile range (i.e., iqr = 25°∼75°), while quantitative results were reported in terms of mean, and standard deviation of the accuracy in “trajectory tracing”, and normalized completion time (i.e., the average velocity to complete the task).

The Kruskal–Wallis test was performed to compare qualitative and quantitative data among groups with different levels of “Experience with AR”/“Experience with HMDs”/“Experience with VST-HMDs”. A *p*-value < 0.05 was considered statistically significant.

## 3. Results

### 3.1. Qualitative Evaluation

Results of the Likert Questionnaire are reported in Table 2. Overall, the participants agreed/strongly agreed with all the statements addressing the ergonomics, the trustability of the proposed AR modality to successfully guide manual task, and confidence on accurately performing the tasks guided by the AR platform. For all questionnaire items, except for item 2 “I perceived VR trajectory as clear and sharp”, and item 7 “The latency of the camera mediated view does not compromise the task execution”, there was no statistically significant difference (*p* > 0.05) in answering tendencies among subjects with different levels of experience with VR, HMDs, and VST-HMDs (see Table 3 for *p*-values). For item 2 and item 7, the agreement level varied according to the expertise of the subject: for item 2, the less experienced participants, namely with no or limited experience with AR and HMDs, and, for item 7, the participants with limited experience with HMDs and VST-HMDs expressed their agreement, whereas the remaining subjects (those with more experience) strongly agreed with both items.

### 3.2. Quantitative Evaluation

**Figure 6:**
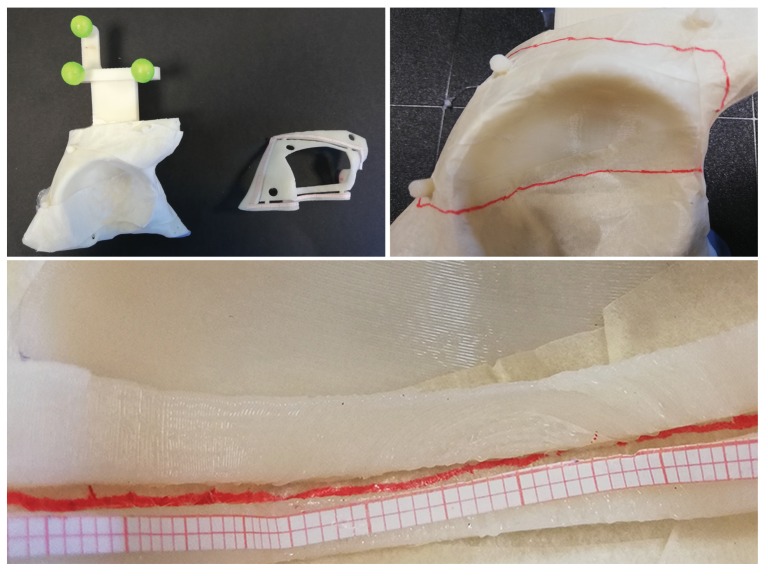
Top Left: 3D printed phantom for T3 with the 0.5 mm accuracy level template. Top Right: Example of a traced trajectory for the T3 task. Bottom: Zoomed detail of the traced T3 trajectory evaluated with the 0.5 mm accuracy level template.

Figure 6 shows an example of traced trajectory for the T3 task. The zoomed detail of the image shows the traced trajectory within the inspection windows of the 0.5 mm accuracy level template. Table 4 summarizes mean and standard deviation values of the accuracy results: for each trajectory (T1, T2, and T3), the subject performance is reported as a percentage of the length of traced line staying within the 0.5 mm and 1 mm accuracy levels. The table reports the success ratio in completing the tasks without committing errors greater than 2 mm: all the subjects successfully completed all the T1 tasks (30/30 success ratio), 9 out of 10 subjects successfully completed all the T2 tasks (29/30 success ratio), and 7 out of 10 subjects successfully completed all the T3 tasks (24/30 success ratio). Overall, all subjects were able to successfully trace the trajectories in at least one of the three trials. Notably, in unsuccessful trials, more than 85% of the traced line was within the 1 mm accuracy level (mean 92±6%).

Table 5 reports performance results in terms of normalized completion time (i.e., the average velocities to complete each task), and shows that on average subjects were slower in completing the T3 trajectory. Mean and standard deviation of the duration of the experiments are reported in the last two columns. Finally, as shown in Table 3, the Kruskal–Wallis test revealed that there were no significant differences (*p* > 0.05) in accuracy performances and normalized completion time between participants with different levels of experience with VR, HMDs, and VST-HMDs.

## 4. Discussion and Conclusions

Recent literature shows that HMDs are emerging as the most efficient output medium to support complex manual tasks performed under direct vision. Despite that, technological and human-factor limitations still hinder their routine use for aiding high-precision tasks in the peripersonal space. In this work, we show the results of a user study aimed to validate a new wearable VST AR platform for guiding complex 3D trajectory tracing tasks.

The quantitative results suggest that the AR platform could be used to guide high-precision tasks: on average, more than 94% of the traced trajectories stayed within an error margin lower than 1 mm and more than 82% of the traced trajectories stayed within an error margin lower than 0.5 mm. Only in 5% of the trials did the users fail in tracing the line having a margin error greater than 2 mm. We can argue that such failures may be due to different reasons, not all of them owing to the AR platform per se but also to the user’s ability. As for the possible source of errors not strictly associated with the AR platform, we noticed that most inaccuracies happened around discontinuities of the phantom surfaces. This may be related not only on a sub-optimal perception of relative depths when viewing through the VST HMD, but also to a more practical difficulty for the user to ensure a firm stroke while following the trajectory over such discontinuities. This is also confirmed by the generally lower velocities experimented in completing the T3 trajectory that is the one on a non-uniform surface.

In this study, the main criterion adopted to select the participants was to include subjects with different levels of experience with VR, HMDs and VST-HMDs, as the 3D trajectory tracing task was general purpose. To apply the proposed AR platform to a more specific industrial or medical application, usability tests with the final users should be performed after having defined, for each specific trajectory tracing task, the most appropriate strategy to track the target 3D surface AR registration strategy. In the field of image-guided surgery, we are currently designing the most appropriate tracking/registration strategy to perform AR-guided orthognathic surgery. In order to properly register the planned 3D osteotomy to the actual patient in the surgical room, we have adopted an innovative patient-specific occlusal splint that embeds the three spherical markers for the inside-out tracking mechanism. For this specific application, we are planning to perform an in vitro study recruiting several maxillofacial surgeons with different level of expertise in orthognathic surgery to test, on patient-specific replicas of the skull, an AR-guided osteotomy of the maxillary bone.

As regards the display (i.e., photon-to-photon) latency caused by the VST mechanism, we have a direct measure of the frame rate of the tracking-rendering mechanism (i.e., ≈30fps yielding a latency of ≈33 ms). For a thorough evaluation of the perceived latency, we must also consider the tracking camera frame rate (i.e., in our system, the camera frame rate is of 60 Hz that produces a latency of 17 ms). Finally, we must also consider the latency caused by the OLED display that contributes with other 17 ms (our HMD runs at 60 Hz). These considerations lead to an overall estimation of the photon-to-photon latency of at least 33+17+17 = 67 ms. Such latency is undoubtedly perceivable by the human vision system. In this study, only a qualitative assessment of latency and spatial jitter/drift due to inaccuracies in the inside-out tracking and to the VST mechanism was performed. However, considering the results obtained with this and previous studies [23,31,32], we can reasonably argue that the proposed wearable VST approach is adequate in ensuring a stable VST AR guidance for manual tasks that demand high accuracy and for which the subject can compensate for display latency by working more slowly.

Even if these results should be confirmed considering a larger number of subjects sample from end users for each specific application, we believe that the proposed wearable AR platform will pave the way for the profitable use of AR HMDs to guide high precision manual tasks in the peripersonal space.

## Figures and Tables

**Table 1:** Demographics of the ten participants to the user study.

General Info	Value
Gender (male; female; non-binary)	(3; 7; 0)
Age (min; max; mean; STD)	(25; 42; 31.9; 6.2)
Visual Acuity (normal; corrected to normal)	(4; 6)
AR experience (none; limited; familiar; experienced)	(1; 3; 2; 5)
HMDs experience (none, limited, familiar, experienced)	(2; 1; 2; 5)
VST HMDs experience (none, limited, familiar, experienced)	(2; 2; 2; 4)

none = technology never used; limited = technology used less than once a month; familiar = technology used about once a month; experienced = technology used several times a month. STD = Standard Deviation; AR = Augmented Reality; HMD = Head Mounted Display; VST = Video See Through.

**Table 2:** Likert Questionnaire results.

Items	Median (iqr)	*p*-Value		
		Exp. with AR	Exp. with HMD	Exp. with VST HMD
I did not experience double vision	4 (3.25∼5)	0.195	0.434	0.158
I perceived VR trajectory as clear and sharp	4.5 (4∼5)	0.029	0.029	0.066
I was able to contemporaneously focus at the VR Content and Real Objects	4 (4∼5)	0.266	0.283	0.753
I was able to clearly perceive Depth relations between VR Content and Real Objects	4 (3.25∼4)	0.167	0.076	0.110
I perceived the VR content pose stable over the time	4 (3∼4)	0.257	0.249	0.226
I did not perceive any visual discomfort due to blur	4 (3∼4)	0.200	0.421	0.499
The latency of the camera mediated view does not compromise the task execution	5 (5∼5)	0.112	0.031	0.031
I did not experience visual fatigue	4 (3∼4.75)	0.249	0.183	0.102
I felt comfortable using this AR guidance modality for the selected task	4 (4∼4)	0.494	0.145	0.337
I can trust this AR modality to successfully guide manual task	4 (4∼5)	0.581	0.160	0.392
I am confident of the precision of manual tasks guided by this AR modality	4 (3.25∼5)	0.535	0.299	0.682

**Table 3:** *p*-values of the Kruskal–Wallis test.

	Exp. with AR	Exp. with HMD	Exp. with VST HMD
Completion Speed	0.501	0.384	0.436
Accuracy Level 0.5 mm	0.951	0.916	0.910
Accuracy Level 1 mm	0.957	0.736	0.663

**Table 4:** Success ratio, mean percentage (μ), and standard deviation (σ) percentage of traced trajectories within the 0.5 and 1 mm accuracy level.

	T1			T2			T3		
Subject	Success	% of Trajectory within		Success	% of Trajectory within		Success	% of Trajectory within	
ID	Ratio	the Accuracy Level		Ratio	the Accuracy Level		Ratio	the Accuracy Level	
		0.5 mm	1 mm		0.5 mm	1 mm		0.5 mm	1 mm
1	3/3	μ=100% σ=0%	100% 0%	3/3	100% 0%	100% 0%	3/3	67% 21%	90% 14%
2	3/3	82% 7%	89% 11%	3/3	88% 7%	100% 0%	3/3	87% 6%	100% 0%
3	3/3	100% 0%	100% 0%	3/3	99% 1%	100% 0%	3/3	81% 3%	91% 8%
4	3/3	90% 10%	100% 0%	3/3	87% 16%	100% 0%	1/3	90% 2%	98% 2%
5	3/3	75% 25%	100% 0%	2/3	83% 16%	91% 8%	3/3	87% 2%	96% 2%
6	3/3	76% 8%	99% 1%	3/3	94% 8%	100% 0%	3/3	84% 10%	89% 11%
7	3/3	77% 40%	100% 0%	3/3	100% 0%	100% 0%	1/3	75% 21%	93% 2%
8	3/3	73% 13%	96% 7%	3/3	100% 0%	100% 0%	1/3	91% 3%	98% 4%
9	3/3	73% 10%	96% 7%	3/3	99% 0%	100% 0%	3/3	88% 5%	94% 6%
10	3/3	74% 5%	100% 0%	3/3	96% 4%	100% 0%	3/3	83% 7%	94% 7%
TOTAL	30/30	82% 17%	98% 5%	29/30	95% 9%	99% 3%	24/30	83% 12%	94% 7%

**Table 5:** Mean and standard deviation of the velocities to complete the tasks.

	T1		T2		T3		Overall Time	
Subject	Mean	Std Dev	Mean	Std Dev	Mean	Std Dev	Mean	Std Dev
ID	[mm/s]	[mm/s]	[mm/s]	[mm/s]	[mm/s]	[mm/s]	[s]	[s]
1	2.8	0.3	3.3	0.5	8.3	2.7	750	83
2	3.5	0.3	3.4	0.5	5.9	0.6	466	41
3	3.0	0.5	2.3	0.2	7.5	1.3	607	48
4	2.2	0.1	2.4	0.5	3.9	0.6	664	72
5	1.9	0.1	1.4	0.1	3.8	0.3	405	33
6	2.4	0.1	2.6	0.5	4.2	0.5	357	35
7	2.2	0.5	3.2	0.6	5.8	1.0	436	35
8	2.6	0.2	3.1	0.4	7.1	2.0	561	52
9	2.1	0.5	1.7	0.2	4.0	1.1	658	69
10	2.5	0.3	2.4	0.4	5.6	1.0	386	38
TOTAL	2.5	0.5	2.6	0.7	5.6	1.9	529	52

## Abbreviations

The following abbreviations are used in this manuscript:

HMD	Head-mounted display
AR	Augmented reality
VST	Video see-through
OST	Optical see-through
